# Towards formal models of psychopathological traits that explain symptom trajectories

**DOI:** 10.1186/s12916-020-01725-4

**Published:** 2020-09-28

**Authors:** Paul B. Sharp, Gregory A. Miller, Raymond J. Dolan, Eran Eldar

**Affiliations:** 1 Max Planck UCL Centre for Computational Psychiatry and Ageing Research, University College London, London, UK; 2 Wellcome Centre for Human Neuroimaging, University College London, London, UK; 3 University of California, Los Angeles, USA; 4 University of Illinois at Urbana–Champaign, Champaign, USA; 5 The Hebrew University of Jerusalem, Jerusalem, IL Israel

**Keywords:** Psychiatric traits, Computational modeling, Bayesian inference, Self-report

## Abstract

**Background:**

A dominant methodology in contemporary clinical neuroscience is the use of dimensional self-report questionnaires to measure features such as psychological traits (e.g., trait anxiety) and states (e.g., depressed mood). These dimensions are then mapped to biological measures and computational parameters.

Researchers pursuing this approach tend to equate a symptom inventory score (plus noise) with some latent psychological trait.

**Main text:**

We argue this approach implies weak, tacit, models of traits that provide fixed predictions of individual symptoms, and thus cannot account for symptom trajectories within individuals. This problem persists because (1) researchers are not familiarized with formal models that relate internal traits to within-subject symptom variation and (2) rely on an assumption that trait self-report inventories accurately indicate latent traits. To address these concerns, we offer a computational model of trait depression that demonstrates how parameters instantiating a given trait remain stable while manifest symptom expression varies predictably. We simulate patterns of mood variation from both the computational model and the standard self-report model and describe how to quantify the relative validity of each model using a Bayesian procedure.

**Conclusions:**

Ultimately, we would urge a tempering of a reliance on self-report inventories and recommend a shift towards developing mechanistic trait models that can explain within-subject symptom dynamics.

## Background

Psychopathology, as conceived dimensionally, is typically assayed in both clinical research and service settings using self-reporting of symptoms. As long as a symptom inventory is shown as reliable in relevant samples and contexts, the instrument is assumed to be sufficient to make inferences that, for instance, an individual has “trait anxiety.” This type of inference commits to a weak, stationary model of that trait: that symptom frequency suffices when denoting an individual is endowed with a given pathological trait. We advance an argument that mechanistic models of what causes symptom fluctuations greatly improve the validity of inferences regarding psychopathological traits by making predictions concerning symptom *trajectorie*s that indicate not only whether, but also when, clinical symptoms are likely to occur and recur.

Pursuing mechanistic models of psychopathology is a core component of an ongoing effort in the psychological sciences to transition from a descriptive to a causal science [1]. This approach seeks to mathematically formalize psychological functions and demonstrate how they are implemented in the human brain [2]. This approach, importantly, does not privilege biological explanations, as full mechanistic models must contain information processing accounts in order to fully understand the dynamics of the relevant biology [3, 4].

Taking such a mechanistic approach, however, is rendered difficult by virtue of assumptions regarding psychopathology, arising out of ingrained practices that measure psychopathological traits via self-report. For instance, mechanistic claims are frequently made by asking individuals to report how often, or for how long, they experience symptoms followed by a mapping of this variance onto some biological dynamics. However, under a simple causal model of depression [5], we show how durable symptoms can arise from two entirely different causes, only one of which would denote a psychopathological trait that predicts future recurrence of said symptoms.

After outlining how a mechanistic model can reveal separate causes for self-report stability, we provide a framework that can help quantify the validity of models that inform how to infer whether an individual has, for example, trait depression. The framework is formalized in a hierarchical Bayesian procedure that validates a trait model by the likelihood it can predict within-subject variation in symptoms that the trait ostensibly gives rise to. The Bayesian procedure states that the probability that the trait model accounts for symptom variation depends on a prior body of evidence for that model.

Ultimately, we derive three practical consequences of adopting this Bayesian framework. First, it constrains scientists and clinicians to be more explicit about the theoretical models supporting the self-report measurement device they use. Secondly, it reveals assumptions behind the use of a self-report measure, whose prior probability of being true can be more clearly evaluated by an examination of the evidence supporting these assumptions. Thirdly, it encourages clinical scientists to become aware of, understand, and help improve upon promising mechanistic models of psychopathology.

## Tacit assumptions of using self-report to measure pathological traits

When we measure trait anxiety, for example, using the common State-Trait Anxiety Inventory [6, 7], what are we actually committing ourselves to? By definition, a trait predisposes an individual to experience certain symptoms. Thus, denoting an individual as having a trait entails a relationship between the trait and within-subject symptom variation, which can be either explicitly or implicitly modeled. Our aim is to shed light on the implicit and explicit models that seek to explain the dynamics relating a trait to within-subject symptom variation. We argue that the typical implicit model is suboptimal at explaining within-subject symptom variation since its predictions are stationary. By contrast, we demonstrate how a computational model of mood can support dynamical predictions and, in our chosen example, better explain the relationship between trait depression and mood variation over time.

We consider trait depression and examine two models that describe how mood can vary over time. The first uses a typical self-report inventory to reveal a trait of depression by querying how often individuals experience classical symptoms of depression, such as anhedonia and depressed mood. We compare this typical model to a computational model of depression that makes different claims about what the trait is, including how it predicts variation in negative mood. Moreover, simulating data using the model demonstrates under what conditions the internal trait is responsible for temporally stable low mood.

## Most self-report trait models do not explain symptom variation within individuals

In describing a common implicit model adopted when measuring traits via self-report inventories, we assume that to denote a subject in a study as having a trait implies that the trait accounts for symptom variation within an individual over time. Below we formalize a class of assumptive models that clinical scientists often adopt when inferring traits from self-report inventories. In this simple regression model, the goal is to predict symptom variation for an individual over a short time period (2 weeks). This implicit model assumes that mood variation is captured via:

1 $$ \mathrm{Mood}=\mathrm{B}1\ast \mathrm{Trait}\ \mathrm{Depression}\ \mathrm{Score}+\mathrm{error} $$

The investigator might also include other variables that predict symptom variation that is not the result of the trait. Therefore, we can expand Eq. (1) to predict symptom variation from the residualized variance of the trait depression score after removing shared variance due to non-trait factors, such as negative major life events (MLE):

2 $$ \mathrm{Mood}=\mathrm{B}1\ast \mathrm{Trait}\ \mathrm{Depression}\ \mathrm{score}+\mathrm{B}2\ast \mathrm{MLE}+\mathrm{error} $$

According to both models, the level of trait depression adds a fixed quantity to mood at any time point, and, thus, it does not predict mood fluctuations.

In addition to the problem that the “stationary” trait models delineated above are likely inaccurate in predicting symptom variation, there is a measurement problem with equating the self-report trait inventory score with the trait itself. The true latent trait is an internal parameter (e.g., genetic, neurobiological, computational) or a set of parameters that accounts for the frequency of symptoms presenting over time. Given that we do not have strong theories relating these stable internal parameters to symptom fluctuation, variance encoded in the self-report data may capture the relevant variance in the true trait highly inaccurately.

This problem is exacerbated by vague queries like, “how often do you experience low mood *in general”* to which one can only respond by choosing a discrete number on an ordinal scale. Indeed, the myriad ways in which individuals recall the rate or frequency of symptom presentation, and then idiosyncratically map this onto the low-dimensional scale, can further obscure the true signal (i.e., the latent trait). This can help explain, for instance, why measures designed to indicate trait negative affect (in general, how often are you sad) have modest test-retest reliability at short time-scales between tests (*r* = 0.65 at 2 months) and even lower reliability across years (*r* = 0.41 at 72 months) [8]. This is mirrored in ostensibly state measures (e.g., Beck’s Depression Inventory [9]) overlapping almost entirely with trait measures (Trait subscale of the State-Trait Anxiety Inventory; *r* = .72) [10].

That said, recent work on self-report vs. task-derived measures of trait constructs has shown that self-report may in some cases yield higher reliability [11, 12]. Validating trait models must include a thorough examination of the reliability of parameters derived from relevant tasks. Indeed, steps in this direction are increasingly evident in computational work [13]. Estimating test-retest reliabilities should also endeavor to capture measurement error inherent to many cognitive tasks [14] which, if not implemented, will diminish estimates of reliability.

More importantly, even if the self-report score is reliably obtained for an individual over time, it may not be a valid index of the relevant level of the trait in question. As such, we intend to demonstrate how an effort to build trait models can be bolstered by *not* relying on trait self-report inventories and instead focusing on other indicators of latent traits (we focus on computational parameters). In principle, the development of trait models can be hindered if the goal is to maximize the covariance between computational parameters and trait self-report data. By contrast, we argue that the validity of trait measures is ultimately determined by a model of such traits capturing within-subject symptom variation over time. Doing so does not necessarily require trait self-report inventories, nor do they necessarily add value to other methods.

## Self-report trait models explaining within-subject symptom variation

Not all models in which traits are measured via self-report assays assume that a trait predicts static symptomatology. For example, models using techniques such as dynamic structural equation modeling and ecological momentary assessment relate traits measured via self-report to within-subject symptom variation [15–17]. Indeed, such models improve upon the ubiquitous implicit model described above in which traits are defined by symptom stability.

However, most self-report trait models lack mechanistic detail. A hypothetical construct measured by self-report, such as trait anxiety, is rarely defined with respect to information processing, making it difficult to reveal how it might be instantiated in the brain and its functional role in larger circuits. By contrast, the computational model of mood variation we advance rests on well-evidenced dynamics of learning and decision-making as implemented in dedicated neural circuits [18].

Taken together, the mere use of self-report assays to measure a trait is not inherently problematic. It is an empirical question as to the extent a given self-report trait measure is predictive of symptom dynamics and generative of novel therapeutic interventions when featured in a trait model. The aim of this paper is to argue for an alternative way of measuring traits and formalizing their role in mechanistic models, an enterprise still in its infancy.

## A mechanistic model of trait depression: learning and mood formalized

A mechanistic model of depression, unlike a self-report approach, specifies a causal system that gives rise to mood. As such, it is an *explicit* trait model. The mechanism is an internal process that takes information from the world and processes it for specific purposes (with complex bidirectional dynamics). Here we focus on a system that learns how valuable different options are in the world in order to inform what are the best decisions. In this model, different aspects, both internal to the mechanism and in the environment, affect what the individual learns about their world, which ultimately influences fluctuations in mood. This process can result in periods of low mood because of internal computational parameters or external dynamics. We denote the former case as an internal trait causing depressive symptoms.

We adopt a pragmatic approach to differentiate psychological states from traits, wherein traits are stable properties over medium to long time scales, whereas states endure for much shorter time scales, such as symptoms of a disease. Importantly, a trait is validated only within a model, wherein a hypothesized stable property of an organism explains variation in symptoms within an individual over time. The model should describe the function of a trait with respect to relevant internal mechanisms, as well as bidirectional influences between these mechanisms and the environment in which an organism is embedded. This conception of a trait is consistent with other functional models of traits, as articulated by [19]. However, we stress that ultimately a mechanistic perspective is needed to describe how a trait is realized in an organism, where this entails a computational understanding of relevant brain mechanisms.

The model we describe and compare to a more typical approach is predicated on developments in computer science that formalize how individuals learn and make decisions. On the surface, these so-called reinforcement learning algorithms may have little to do with mood, but a body of evidence suggests otherwise. Momentary happiness [20] is predicted well by fluctuations in variables involved in decision-making algorithms, while more recently a classical reinforcement-learning process has been extended to model how mood is directly computed from what is learned [21].

Below we present a computational model of depressed mood that is built on these learning algorithms. The model is instantiated in two individuals whose parameters within the model are set to different levels. We highlight which of these internal parameters can be thought of as the relevant trait conferring risk for depression. This trait becomes predictive of mood variation within subjects when situated within a sufficiently described set of dynamics of the internal cognitive process and the environment in which the individual is situated. As such, an explicit trait model comprises the trait (internal parameter), the mechanism within which the trait resides (e.g., modified model-free reinforcement learning) and the environment affecting the dynamics of the mechanism (e.g., the pace and levels of reward).

In this example, consider two individuals who take a new job and must learn how trustworthy two new colleagues they will work closely with are. Each time they interact with one of these new colleagues, they receive feedback they can use to update the trustworthiness of the colleague. The trustworthiness goes up when they see their colleagues responding in line with their expectation, and it goes down when the colleague ignores or acts against the expectation. The overarching goal is to learn which colleague is more trustworthy, so they can avail themselves of the more reliable relationship. This can be modeled using simple learning equations.

3 $$ {\mathrm{Trust}}_{c,t}={\mathrm{Trust}}_{c,t-1}+{\eta}_{\mathrm{positive}}\left({\delta}_t\right) $$

4 $$ {\mathrm{Trust}}_{c,t}={\mathrm{Trust}}_{c,t-1}+{\eta}_{\mathrm{negative}}\left({\delta}_t\right) $$

5 $$ {\delta}_t={\mathrm{Outcome}}_t-{\mathrm{Trust}}_{c,t-1} $$

Trust in a colleague (c) is an internal variable that in practice is inferred from the colleague’s behavior and is updated over time (*t*) according to Eqs. 1 and 4. The previous level of trustworthiness for a given colleague (c) either increases or decreases after choosing to interact with them. This update depends on how different the colleague’s response (outcome at time *t*) is from how one expects that colleague to respond (the value of Trust at *t* − 1). The difference between the actual response and the expected response (called the “prediction error”) is highlighted in Eq. 5. Again, the variable Trust represents how often one expects the colleague to respond in line with one’s request, such that the greater the trustworthiness, the more one expects a colleague to respond well. The *η* parameters that multiply this prediction error determines *to what degree* one’s trustworthy estimate for the colleague is updated based on the most recent prediction error. When *η* is small, expectations are updated slowly. When it is large, they are updated rapidly. *η* is bounded between 0 and 1, where 0 reflects no updating and 1 reflects that a new trustworthiness value takes into account only the most recent prediction error.

6 $$ {\mathrm{Mood}}_t={\mathrm{Mood}}_{t-1}+{\phi}_{\mathrm{mood}}\left({\delta}_t-{\mathrm{Mood}}_{t-1}\right) $$

The reason that there are two different *η* parameters, differentiating Eqs. 1 and 2, is that this model asserts that the update of one’s trust in a colleague depends on whether one is positively or negatively surprised. When one’s expectation is *lower* than the most recent outcome (positive surprise), that prediction error updates one’s estimate *at a different rate* than if one’s expectation was *greater* than the most recent outcome (negative surprise). As will be shown, the difference between these two update rates, known more commonly in the computational literature as “learning rates”, can result in depressive symptoms.

How does this all relate to mood? As specified in Eq. 6, current mood is a function of how surprised one has been in the recent past. That is, mood goes up when one experiences a series of positive surprises, and the opposite is the case when one experiences negative surprises. The expected amount of surprise is updated by its own learning rate, ɸ. Surprise, or more technically, the “prediction error”, has been shown to relate to fluctuations in mood over short [20] and long [22] time scales. Situating this in the context of the running example, one’s mood will be low to the extent one experiences surprisingly poor outcomes from one’s colleagues. We stick to this simple model, but in principle, these learning rules, and the mood computed from the outcomes of these decisions, can be generalized over many decision domains that capture more realistically how mood is related to an amalgam of decisions in one’s daily life.

7 $$ P\left(\mathrm{NextDecision}|c1\right)=\frac{{{}_e}^{{\mathrm{Trust}}_{c1}}}{{\sum_{ce}}^{{\mathrm{Trust}}_c}} $$

Finally, when one faces a decision about which colleague to interact with, the decision is made in accordance with Eq. 7. Here, the probability one interacts with the first colleague, which we call “c1”, is determined by its proportion of the total trustworthiness of all colleagues with the slight caveat that each trustworthiness is scaled exponentially. All probabilities for interacting with colleagues are derived in this in this way, and the resultant categorical distribution is then randomly sampled from to determine which colleague one interacts with. Parameters can be added that relate how willing one is to explore interacting with less trustworthy colleagues, which is advantageous when one has not accrued much experience with them. We focus less on this decision process, as it is more centrally the learning rules that govern how mood fluctuates that is of present interest.

## Simulating how persistent low mood can result from intrinsic traits and external causes

We now present simulations of two individuals in two different work environments. One individual has what we denote as trait depression, defined by a greater learning rate for positive surprises than for negative surprises. This sounds counter-intuitive, but we consider depression emerges as a function of more slowly learning about negative outcomes [23]. That is, the individual fails to update their expectations about negative events and therefore believes their co-workers to be more trustworthy than they truly are. In doing so, their elevated expectations lead to disappointment in the form of negative surprises, which accumulate over time to account for more prolonged periods of low mood. The second individual, designated “healthy” in the figure, has no bias to experience depressed mood due to an imbalance in the learning process. However, the healthy individual can exhibit the same prolonged period of depressed mood when in a “bad environment” in which they are repeatedly treated poorly.

**Fig. 1:**
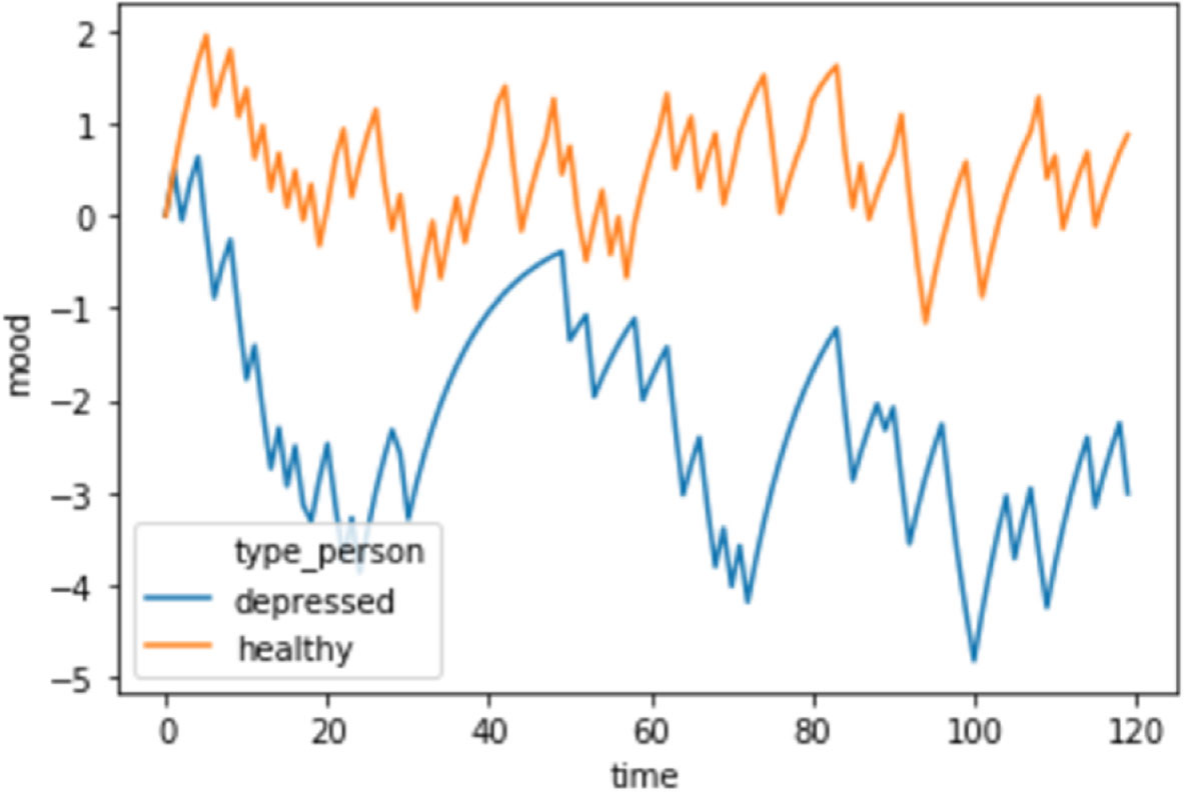
Two individuals in a relatively good work environment. Time on the *X* axis is in weeks, whereas mood is a function of the running weighted average of surprise (e.g., average negative surprise relates to low mood). A depressed agent (blue line) learns much more from positive surprises than negative surprises and, as a consequence, is set up for chronic disappointment. This fosters persistent low mood and can be thought of as an instantiation of “mood-reactive depression” [24]

When the depressed agent interacts with two colleagues who are somewhat trustworthy (one 40% of the time, the other 70% of the time), their mood can go through periods of extended depression due to learning much more from positive than from negative surprise. This imbalance in learning rate means that their estimates of the trustworthiness of colleagues are overly optimistic, to a degree that they are setting themselves up for disappointment. For this example, we define a significantly low mood as less than − 2 on the scale of mood in Fig. 1. If time is measured in weeks, then there are periods in which the depressed individual has low mood for several weeks. By contrast, a “healthy” individual interacts with the same two individuals but possesses the same learning rate for both positive and negative surprises. Figure 1 indicates that they never endure long periods of depressed mood.

**Fig. 2:**
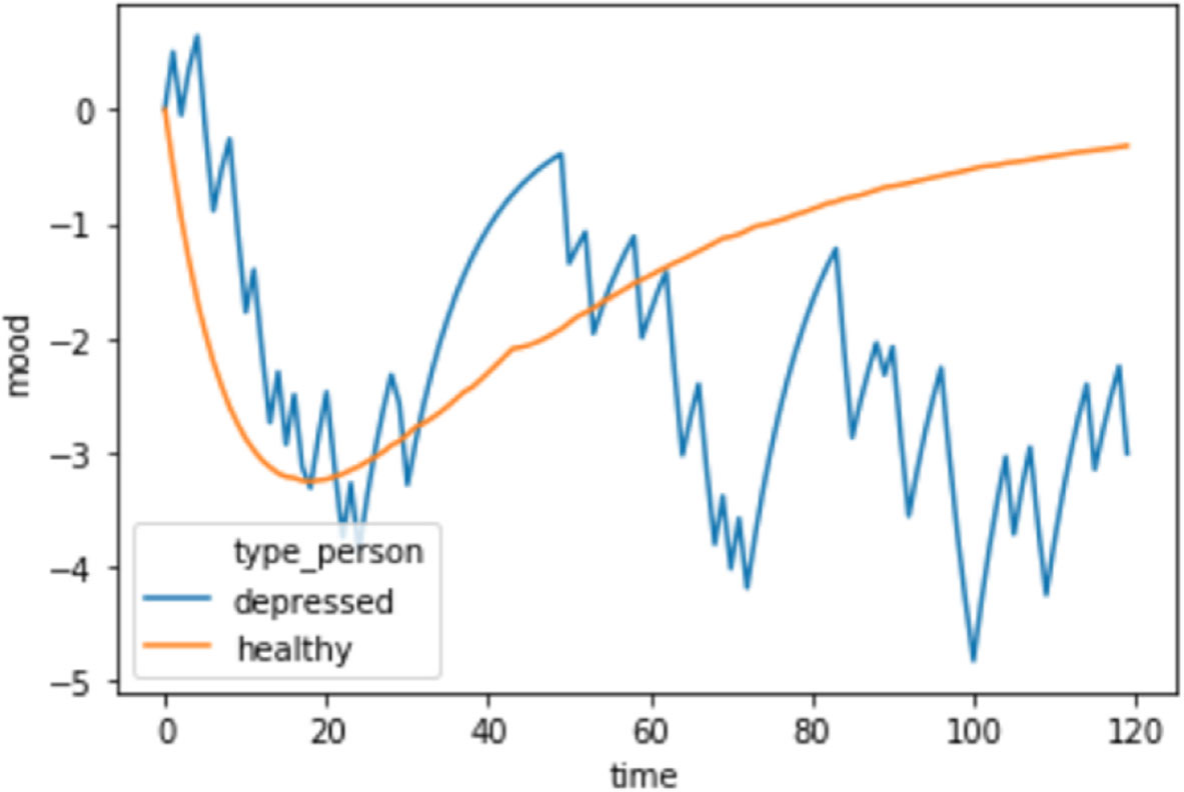
A healthy individual in a bad environment compared to a depressed individual in a good work environment. The time series for a depressed individual (blue line) is *the same* as in Fig. 1. Again the *X* axis is on the scale of weeks, whereas the *Y* axis represents mood as a function of surprise. A healthy individual (orange line), by contrast, learned about their colleagues in a terrible work environment. As such, their initial dip in mood follows closely that of a depressed individual in a healthy environment, but they recover

In Fig. 2, we compare the same depressed individual in Fig. 1 who works in a relatively good work environment to a healthy individual in a bad work environment. The bad work environment is a function of the colleagues the healthy colleague interacts with being highly untrustworthy (95% and 98% of the time). As a result of this bad environment, from week 3 to week 40, the healthy individual experiences a persistent low mood. If this individual were queried with a trait self-report measure of depression, they may answer such that they qualify as having trait depression. However, in the present framework, given their learning-rate parameters, they do not possess the internal trait (mechanistically, difference in learning from positive and negative surprises) of depressive symptoms that predicts the recurrence of low mood. One might argue that requiring high test-retest reliability prevents such false positives from substantially impacting one’s self-report assay. However, the time period of test-retest likely bears heavily on these inferences, as does taking account of the likelihood that environmental influences can be *persistent* over time.

Furthermore, although the focus has been on latent computational traits predicting within-subject variation, they, too, affect between-subject differences in mood. For example, in the relatively normal working environment (Fig. 1), having the depressive trait results in both a lower mean and higher standard deviation of mood. This comports with latent trait models of psychopathology that suggest that traits affect both first and second moments of symptom distributions [25].

## Probabilistic inferences about traits depend on model validity

A probabilistic inference regarding the level of trait depression an individual possesses should be adjusted by evidence. To do so would require a formal model comparison procedure that defines a suitable target dataset that the model can account for. Moreover, the comparison procedure should be cumulative, aggregating across studies that attempt to model the relevant data. Below, we formalize a hierarchical Bayesian procedure that fits trait models to within-subject symptom variation. Moreover, the model^1^ itself depends on the prior body of evidence regarding its ability to explain relevant symptoms.

8 $$ p\left(\mathrm{SymptomData}|\mathrm{model}\right)=\int d\theta P\left(\mathrm{SymptomData}|\mathrm{model},\theta \right)P\left(\theta |\mathrm{model}\right) $$

In the above, *θ* refers to the parameters of the trait model, which for instance could be an individual’s learning rates. To derive validity for the model, also called model evidence, the equation averages all possible individual instances of the model (i.e., all the kinds of individuals with different individual parameters that could be utilizing that model).

Thus, to calculate the validity of the trait model requires collecting data from many subjects that ensures sufficient within-subject data on the relevant dynamics to which the hypothetical trait predisposes an individual. In the example above, we simulated far fewer interactions than those that present the totality of interactions with colleagues. Moreover, there are likely other types of interactions between the person and aspects of their environment (other individuals, other states and outcomes) that, if modeled, may improve model validity.

Even though it is not possible to exhaust the measurement of the relevant factors influencing symptoms dynamics, two generative approaches may help. The first is to collect and model relevant real-life interactions when possible; indeed, this requires more intensive sampling of non-laboratory daily-life dynamics than is typical currently in attempts to formally model symptom variation computationally. The second is to obtain a weighted average over all possible life events that are relevant and go unmeasured, along with assumptions regarding how these factors impact the relevant symptom dynamics. When possible, these assumptions should be encoded as empirical priors.

Although the field has only begun to model symptom variation with computational models, there are now good examples of success in modeling ecologically valid temporal patterns of symptom variation in daily life. For instance, [22] mapped mood variation within subjects to physiological signals generated by two co-occurring learning systems that internalize statistical quantities about rewards along different timescales. Moreover, large-scale smartphone-based approaches have validated models of decision-making with varying levels of happiness in daily life [26].

## The pervasiveness of the “self-report=trait” model

The problem identified regarding a reliance on self-report as indices of latent traits impacts much research at the forefront of mechanistic model development, which we, like most in the field of cognitive and computational neuroscience, frequently rely upon. That is, it is often the case that computational parameters, such as those defined above (learning rates for positive and negative surprises), are mapped onto psychological traits derived from self-report. This practice de facto treats self-reports as the gold-standard measure of a trait, possibly hindering a potential for discovery of alternative, more powerful, measures.

We have endeavored to demonstrate how manifest symptoms measured by the self-report device may, but equally may not, be the expression of a relevant latent trait. State measures of depressed mood like the Beck’s Depression Inventory [9] may fail to detect the trait because, as shown in a mood variation model, low mood is not omnipresent. Similarly, a trait depression measure is often accompanied by an implicit model of mood variation that cannot explain dynamic fluctuations that exist *even* when an individual experiences “persistent low mood.” Moreover, similarly high scores on the trait depression inventory for different individuals may have different meanings, either because they map differently to the latent computational trait, or due to the difficulty in accurate reporting which can result in false positives.

These validity issues are not unique to trait self-report inventories. Additional assumptions in computational models might be equally invalid. For instance, the model above assumes that learning rates for positive and negative surprise are stable across time. The ultimate test of whether or not this is veridical is to compare models where these parameters are either time-varying or not, wherein the former might predict under what conditions they change. However, this serves again to emphasize a strength of a formal modeling approach, which can iteratively update model validity via a model’s ability to explain within-subject symptom variation.

## Explaining symptom variation via mechanisms can inform a network approach

The network approach to modeling psychopathology addresses some of the concerns laid out here, not by proposing models of latent traits, but by proposing and testing causal models of symptoms. On the surface, this seems incompatible with the approach offered here. However, we regard both approaches as part of a large family of causal models of symptoms, which, if appropriately designed, can be fit to individual symptom trajectories. Thus, we argue that the two approaches can work in tandem to further elucidate how traits feature in within- and between-subject models of symptom dynamics.

More specifically, we propose that the network approach can be strengthened by mechanistic models of internal processes in computational neuroscience. For instance, in a network model of panic attacks [27], the symptom of “perceived threat” has a causal and dynamic relationship with levels of arousal. Although this symptom is indicated via self-report, the phenomenon of perceived threat can be explained more deeply by parsing the process of how we designate stimuli as threatening based on experience. For example, contextual cues previously associated with threat might bias sampling from one’s memory to evaluate whether a current stimulus is threatening [28]. Such models could infer changing contextual influences on perceived threat from trajectories of choices and thus provide greater insight into the underpinnings of perceived threat than those offered by self-report alone. Such integration of mechanistic models with network approaches can add dynamical predictions regarding symptom trajectories, which some network models (unlike [27]) lack. Moreover, computational theories can spur advances in testing causal claims (for example converting a computational model to a directed acyclic graph in line with Pearl [29]) in regard to what aspects of one’s internal environment may affect symptom patterns.

## Conclusion

Inferences about psychopathological traits are inextricably tied to models of those traits, and we argue that the standard approach serves to hinder further model development. Indeed, examples of this dominant approach pervade areas of psychological, neuroscientific, and computational approaches to cognition. We highlight the benefits from formalizing trait models that allow prediction of within-subject symptom trajectories and show how such models can be evaluated using a Bayesian model comparison approach.

## Data Availability

No data was collected for the present Opinion paper.

## Footnotes

1 Importantly, the model above can be thought of as an abstraction of the cognitive process relating learning and decision-making to mood. The parameter values for theta define specific instantiations of the model. When the model is fit to individual subject data, some or all of the parameters (in the running example here, learning rates for positive and negative surprises) that best account for their data can be thought of as approximations to the latent trait. However, both the model and its parameters are not equivalent to the cognitive process actually implemented in an individual.
